# Whole-genome linkage analysis in mapping alcoholism genes using single-nucleotide polymorphisms and microsatellites

**DOI:** 10.1186/1471-2156-6-S1-S28

**Published:** 2005-12-30

**Authors:** Shuang Wang, Song Huang, Nianjun Liu, Liang Chen, Cheongeun Oh, Hongyu Zhao

**Affiliations:** 1 Department of Biostatistics, Mailman School of Public Health, Columbia University, New York, NY 10032, USA; 2 Program of Computational Biology and Bioinformatics, Yale University, New Haven, CT 06520, USA; 3 Department of Epidemiology and Public Health, Yale University, New Haven, CT 06520, USA; 4 Department of Biostatistics, University of Alabama at Birmingham, Birmingham, AL 35294, USA; 5 Department of Molecular, Cellular and Developmental Biology, Yale University, New Haven, CT 06520, USA; 6 Division of Biostatistics, Department of Preventive Medicine, University of Medicine and Dentisry of New Jersey, Newark, NJ 07101, USA; 7 Department of Genetics, Yale University, New Haven, CT 06520, USA

## Abstract

There is currently a great interest in using single-nucleotide polymorphisms (SNPs) in genetic linkage and association studies because of the abundance of SNPs as well as the availability of high-throughput genotyping technologies. In this study, we compared the performance of whole-genome scans using SNPs with microsatellites on 143 pedigrees from the Collaborative Studies on Genetics of Alcoholism provided by Genetic Analysis Workhsop 14. A total of 315 microsatellites and 10,081 SNPs from Affymetrix on 22 autosomal chromosomes were used in our analyses. We found that the results from the two scans had good overall concordance. One region on chromosome 2 and two regions on chromosome 7 showed significant linkage signals (i.e., NPL ≥ 2) for alcoholism from both the SNP and microsatellite scans. The different results observed between the two scans may be explained by the difference observed in information content between the SNPs and the microsatellites.

## Background

There is currently great interest in using SNPs in genetic linkage and association studies because of the abundance of SNPs as well as the availability of high-throughput genotyping technologies. Kruglyak [1] predicted in a theoretical study that maps with approximately two to three times the density of SNPs with a heterogeneity of 0.5 would be equivalent to the current microsatellites maps. With current high-throughput SNP genotyping technologies, it is now feasible and affordable to collect genotype data from tens of thousands of SNPs. John et al. [2] described the first whole-genome scans with linkage analysis of a complex disease, rheumatoid arthritis, to compare SNPs with microsatellites directly. In this paper, using the Collaborative Studies on Genetics of Alcoholism (COGA) data provided by Genetic Analysis Workshop 14 (GAW14), we compared the results based on whole-genome scans of 143 pedigrees using 315 microsatellites and 10,081 SNPs from Affymetrix across 22 autosomal chromosomes.

## Methods

### Nonparametric linkage analysis

COGA data provided by GAW14 include 143 pedigrees with 1,614 individuals genotyped with both microsatellites and SNPs. In addition, the genetic maps for both the microsatellites and the SNPs were provided. We used the nonparametric linkage analysis implemented in MERLIN [3] for linkage analysis. Individuals were defined as unaffected with alcoholism if they never drank alcohol or if they showed some alcohol-related syndromes but did not meet the criteria for alcoholism [4]. Allele frequencies were estimated using all genotyped individuals, and the Whittemore and Halpern "ALL" statistic [5] was applied for the scan procedure, in which the NPL scores based on all affected pedigree members were calculated. Both the SNP scan and the microsatellite scan were performed at each marker locus.

### Genotyping error detection

To avoid potential bias caused by possible genotyping errors on linkage signals, the error-checking algorithm implemented in MERLIN was applied. This algorithm identifies unlikely genotypes based on the inferred double recombination events, when erroneous genotypes can imply excessive and unlikely recombination events between tightly linked markers [3]. We used the default parameter in MERLIN, where the likelihood ratio of an erroneous genotype with *p *≤ 0.025 was excluded [2]. The two whole-genome scans were carried out both with and without the erroneous genotypes to exam the effect of genotyping error on the scan results.

### Information content (IC)

The major advantage of using high density SNPs versus microsatellites is the increased information content (IC). IC was calculated using MERLIN to compare the microsatellites and the SNPs in order to investigate factors contributing to the differences between the two scans. The microsatellites were spaced an average of 13 cM apart, whereas the SNPs were spaced an average of 0.35 cM apart. To assess the effect of the reduced IC on the SNP scan, a 3,360-SNP map with an average spacing of 1.0 cM was randomly extracted from the full set of SNPs as a subset for a separate scan.

## Results

### Nonparametric linkage analysis

**Figure 1:**
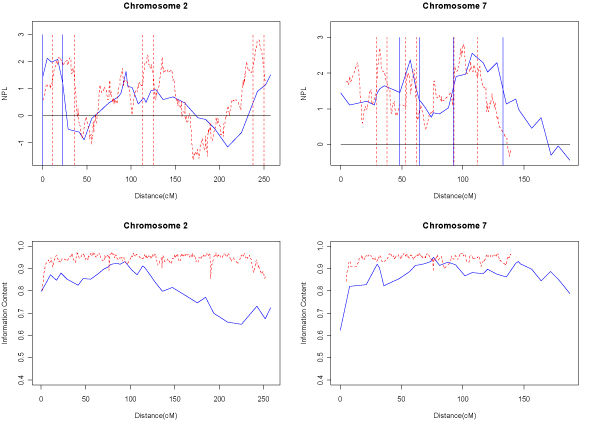
**Multipoint nonparametric linkage scores and IC from 1-cM SNP scan for chromosomes 2 and 7**. Blue solid line, microsatellites; red dashed line, SNPs. Vertical lines, 1-LOD intervals. IC, information content. Erroneous genotypes were excluded from the analyses.

The results from the whole-genome scans using the microsatellites and the SNPs had good overall concordance. Six regions showed some evidence of increased allele sharing, with a NPL cutoff value of 2 for either the SNP scan, the microsatellite scan, or both. The results were summarized in Table 1, which also included analyses containing erroneous genotypes. Overall, the scan using the SNPs gave stronger linkage signals than those using the microsatellites. Except for two regions on chromosomes 2 and 13 that showed significant linkage evidence using the microsatellites but not using the SNPs (there was no SNP genotyped in the region on chromosome 13), the SNP scan gave stronger linkage signal. Four regions on chromosomes 1, 2, 12, and 13 showed significant linkage evidence when using the SNPs but not using the microsatellites. Both the SNP and the microsatellite scans indicated strong linkage signals on chromosome 7, and relatively strong linkage signals on chromosome 2. Results for these two chromosomes (excluding the erroneous genotypes) and one-LOD confidence intervals of these peaks are shown in Figure 1. In general, the peaks were better defined by the SNP scan, where peaks from the SNP scan had narrower 1-LOD intervals than those from the microsatellite scan (SNP 1-LOD interval was 20 cM, compared with a 40-cM 1-LOD interval with the microsatellites for the peak on chromosome 7 around 100 cM. For the peak on chromosome 7 around 60 cM, the SNP 1-LOD interval was 9 cM, compared with a 16-cM 1-LOD interval with the microsatellites. One-LOD intervals for the peaks on chromosome 2 around 10 cM had similar width for the SNPs and the microsatellites.) The NPL scores decreased in the SNP 1-cM scan for all but one region on chromosome 2 at about 18 cM compared to those from the SNP full set scan. With the NPL cutoff of 2, several regions on chromosomes 2, 7, and 12 that were significant in the SNP full set scan were no longer significant in the SNP subset scan. We also noted that the effect of genotyping error on the linkage results was small for this particular data set, although potential genotyping errors seemed to increase the linkage signal slightly, which contradicted with the finding of John et al. [2], who suggested that removal of unlikely genotypes could increase the significance of nominal loci. The discrepancy may due to the different genotyping error rates in the two data sets. There were 1,295 microsatellite genotypes that were likely to be errors and were set missing with MERLIN's error checking algorithm. Among the 1,614 individuals and 315 microsatellites, there were a total of 353,015 genotypes, so the error rate for the microsatellite markers was estimated to be 0.367%. Similarly, there were 27,338 SNP genotypes that were likely to be errors and were set missing with MERLIN's error checking algorithm. The error rate for the SNPs was estimated to be 0.204% as among the 1,614 individuals and 10,081 SNPs, there were a total of 13,395,832 genotypes.

### Information content (IC)

The mean IC for each individual chromosome for the full SNP set, SNP subset, and microsatellites across 22 autosomal chromosomes when the erroneous genotypes were either excluded or included were summarized in Table 2. The IC for the full SNP set was significantly and uniformly higher than that for the microsatellites. When the erroneous genotypes were excluded, the mean genome-wide IC for the microsatellites was 0.783, with an inter-quartile range of 0.134, and was 0.950 for the SNPs with an inter-quartile range of 0.017. The mean IC for the SNP subset was 0.905 with an inter-quartile range of 0.044 when erroneous genotypes were excluded. When erroneous genotypes were included, the mean IC for the SNP subset was 0.910 with an inter-quartile range of 0.042. We noted that although genotyping errors were expected to reduce the values of IC slightly, their impact was quite small, which may be due to the small genotyping error rate.

## Discussion

We have compared the genome-wide linkage analyses based on the microsatellites and the SNPs. We used the software MERLIN to conduct nonparametric linkage analysis to map regions associated with alcoholism on 22 autosomal chromosomes. The results from the two scans had good concordance in general, although more significant signals were obtained using the SNPs versus the microsatellites. Both scans suggested strong linkage evidence on chromosomes 2 and 7, where the two scans agreed especially well. The microsatellite scan had a peak at the marker D7S820 at 107.5 cM with an NPL score of 2.56 on chromosome 7, and the SNP scan had a peak at the marker tsc0046246 at 100.9 cM with an NPL score of 2.81. For chromosome 2, the microsatellite scan had a peak at the marker D2S1329 at 4.9 cM with an NPL score of 2.13, and the SNP scan had a peak at the marker tsc0056805 at 243.6 cM with an NPL score of 2.80. The differing results observed in the two scans were likely explained by the difference between the IC in the microsatellites and the SNPs. In fact, the higher IC is one major advantage of the high-density SNPs compared with the conventional microsatellite sets. The IC across the genome for the SNPs was uniformly higher than that for the microsatellites.

As expected, the analysis based on the SNP subset showed decreased IC and reduced linkage signals compared with the SNP full set, which suggested that the difference in IC might be one key factor that contributed to the observed difference in the two scans. This was consistent with the conclusion from John et al. [2], who examined possible reasons for the observed difference between the scans using the SNPs and the microsatellites comprehensively, including the genotyping errors of the SNPs and the microsatellites, the possible errors in the two maps used, the presence of linkage disequilibrium (LD), and the differences in IC. We have also investigated the possible effect of genotyping errors on the linkage results. Out results suggested that the impact of genotyping errors was quite small for the COGA dataset, which may be due to the small genotyping error rate (0.37% for the microsatellites and 0.20% for the SNPs) and may not be generalized to other data sets. It is worth noting that for the full SNP set with an average spacing of 0.35 cM, it is highly possible that there is LD between SNPs, which may influence the linkage results from MERLIN since MERLIN assumes linkage equilibrium between all markers. John et al. [2] explored the possible effect of LD on the two scans by keeping one SNP from a group of SNPs in LD, or by assigning haplotypes to individuals for clusters of SNPs in LD and treating them as multi-allelic markers. They found that for both cases, there were losses in IC, which made it difficult to assess whether the difference observed in the two scans were due to LD or to losses in IC. They concluded that overall the results were qualitatively similar when SNPs in LD were included or excluded.

Finally, we noted that the SNP subset scan was able to detect some regions detected by the SNP full set scan, and the SNP subset had an average IC of 0.910 compared to the average IC of 0.950 for the full SNP set. With the NPL cutoff of 2, the SNP subset scan resulted in some loss of significance of several regions on chromosomes 2, 7, and 12.

## Conclusion

We have identified two regions that showed some evidence of linkage with alcoholism on chromosome 2 and chromosome 7 from both the microsatellite and the SNP scans. For these regions, we had stronger linkage signals using the SNPs than those using the microsatellites. Although results from the two scans had good overall concordance, three regions of significant linkages were detected in the SNP scan but not in the microsatellite scan. Lastly, the difference in IC between the SNPs and the microsatellites might explain the different results observed in the two scans.

## Abbreviations

COGA: Collaborative Study on the Genetics of Alcoholism

GAW: Genetic Analysis Workshop

IC: Information content

LD: Linkage disequilibrium

SNP: Single-nucleotide polymorphism

## Authors' contributions

SW participated in the design of the study, performed the analysis, and drafted the manuscript. SH, NL, LC, and CO participated in the design and the discussion of the study. HZ conceived the study and helped to draft the manuscript. All authors read and approved the final manuscript.

## Figures and Tables

**Table 1:** Regions that show some evidence of increased allele sharing. Results shown are when NPL scores are greater or equal to 2.0 on either SNP scan or microsatellite scan or both with and without erroneous genotypes.

		Microsatellites (excluding erroneous genotypes) (*n *= 315)		SNPs from Affymetrix (excluding erroneous genotypes) (*n *= 10,081)		SNPs 1 cM subset (excluding erroneous genotypes) (*n *= 3,360)		Microsatellites (with erroneous genotypes (*n *= 315)		SNP (with erroneous genotypes (*n *= 10,081)		SNPs 1 cM subset (with erroneous genotypes) (*n *= 3,360)	
							
Chr.	Position (cM)	NPL score	*P*	NPL score	*P*	NPL score	*P*	NPL score	*P*	NPL score	*P*	NPL score	*P*
1q	77	0.24	0.4	1.80	0.04	1.64	0.05	0.29	0.4	2.06	0.02	2.10	0.02
	146	0.85	0.2	1.97^a^	0.03	1.88^a^	0.03	0.82	0.2	2.06	0.02	1.63	0.05
													
2q	5	2.13^a^	0.02	1.14	0.13	0.52	0.3	2.19	0.014	1.29	0.10	0.27	0.4
	18	2.08	0.02	2.16	0.02	2.35^h^	0.009	2.08	0.02	2.19	0.014	2.29^h^	0.01
	118	0.49	0.30	2.24	0.013	1.88	0.03	0.54	0.30	2.00	0.02	2.13^i^	0.02
	135	0.59	0.30	2.15	0.02	1.83	0.03	0.29	0.40	2.03	0.02	1.67	0.05
	244	0.90	0.20	2.80^a^	0.003	2.46^a^	0.007	0.92	0.20	3.04	0.0012	2.46	0.007
													
7q	14	1.21^b^	0.11	2.30	0.011	1.77	0.04	1.64^b^	0.05	2.30	0.011	1.66	0.05
	32	1.56	0.06	2.69	0.004	2.36	0.009	1.83	0.03	2.73	0.003	2.32	0.01
	60	2.37^c^	0.009	2.10	0.02	1.32	0.09	2.83^c^	0.002	2.02	0.02	1.30	0.1
	94	1.90	0.03	2.20	0.014	2.01	0.02	2.28	0.011	2.20	0.014	1.92	0.03
	101	1.97	0.02	2.81^a^	0.002	2.51^a^	0.006	2.10	0.02	2.88	0.002	2.47	0.007
	106	2.56^a^	0.005	2.32	0.01	1.94	0.03	2.45	0.007	2.32	0.01	2.07	0.02
													
11q	107	1.32	0.09	2.24^a,f^	0.012	2.14^a^	0.02	1.32	0.09	2.23	0.013	2.15	0.02
	120	2.61^a^	0.004	NA^g^	NA	NA	NA	2.60	0.005	NA	NA	NA	NA
													
12q	122	1.02^d^	0.2	2.02^a^	0.02	1.87^a^	0.03	1.02^d^	0.2	1.95	0.03	1.81	0.04
													
13q	86	1.15^e^	0.13	2.63^a^	0.004	2.61^a^	0.005	1.31^e^	0.10	2.56	0.005	2.55	0.005

^a^Peak location on that chromosome

^b^At position 21

^c^At position 57

^d^At position 117

^e^At position 90

^f^At position 108 with NPL score 2.40

^g^NA, not available

^h^At position 20

^i^At position 120

**Table 2:** Mean information content and its standard deviations across 22 autosomes. Results shown are for SNP full set, 1 cM SNP subset, and microsatellites.

Chr	Mean information content (SD)					
	
	SNP (excluding erroneous genotypes)	SNP (including erroneous genotypes)	Microsatellites (excluding erroneous genotypes)	Microsatellites (including erroneous genotypes)	SNP 1 cM subset (excluding erroneous genotypes)	SNP 1 cM subset (including erroneous genotypes)
1	0.951 (0.020)	0.947 (0.020)	0.821 (0.073)	0.821 (0.072)	0.914 (0.040)	0.909 (0.045)
2	0.955 (0.017)	0.952 (0.017)	0.831 (0.082)	0.830 (0.081)	0.918 (0.040)	0.914 (0.041)
3	0.954 (0.016)	0.951 (0.016)	0.745 (0.086)	0.745 (0.086)	0.917 (0.042)	0.914 (0.044)
4	0.953 (0.028)	0.949 (0.028)	0.763 (0.036)	0.761 (0.036)	0.921 (0.046)	0.916 (0.055)
5	0.954 (0.018)	0.951 (0.019)	0.791 (0.078)	0.789 (0.078)	0.916 (0.043)	0.913 (0.046)
6	0.954 (0.017)	0.951 (0.016)	0.784 (0.080)	0.782 (0.079)	0.921 (0.031)	0.918 (0.033)
7	0.953 (0.014)	0.950 (0.015)	0.879 (0.062)	0.878 (0.061)	0.916 (0.031)	0.912 (0.033)
8	0.954 (0.016)	0.950 (0.017)	0.801 (0.079)	0.800 (0.078)	0.916 (0.033)	0.911 (0.039)
9	0.956 (0.020)	0.951 (0.020)	0.763 (0.088)	0.764 (0.089)	0.916 (0.042)	0.911 (0.045)
10	0.953 (0.015)	0.950 (0.015)	0.662 (0.084)	0.662 (0.084)	0.917 (0.036)	0.914 (0.040)
11	0.936 (0.017)	0.933 (0.019)	0.699 (0.054)	0.698 (0.055)	0.910 (0.035)	0.903 (0.046)
12	0.952 (0.021)	0.947 (0.023)	0.807 (0.086)	0.806 (0.086)	0.906 (0.062)	0.899 (0.066)
13	0.951 (0.027)	0.948 (0.027)	0.791 (0.092)	0.792 (0.092)	0.916 (0.056)	0.913 (0.057)
14	0.947 (0.032)	0.942 (0.032)	0.762 (0.048)	0.761 (0.047)	0.909 (0.050)	0.902 (0.054)
15	0.949 (0.017)	0.945 (0.017)	0.801 (0.032)	0.801 (0.033)	0.907 (0.038)	0.903 (0.038)
16	0.937 (0.045)	0.933 (0.045)	0.738 (0.089)	0.737 (0.088)	0.877 (0.077)	0.867 (0.093)
17	0.930 (0.050)	0.926 (0.049)	0.705 (0.082)	0.704 (0.082)	0.859 (0.061)	0.851 (0.073)
18	0.949 (0.017)	0.945 (0.017)	0.678 (0.040)	0.678 (0.039)	0.895 (0.049)	0.889 (0.050)
19	0.902 (0.074)	0.899 (0.073)	0.709 (0.041)	0.710 (0.042)	0.759 (0.142)	0.747 (0.149)
20	0.941 (0.028)	0.936 (0.029)	0.750 (0.115)	0.750 (0.115)	0.879 (0.062)	0.873 (0.067)
21	0.948 (0.026)	0.945 (0.026)	0.780 (0.074)	0.780 (0.075)	0.899 (0.043)	0.892 (0.053)
22	0.904 (0.046)	0.901 (0.046)	0.644 (0.131)	0.644 (0.130)	0.794 (0.101)	0.786 (0.102)
Overall	0.9500 (0.025)	0.9465 (0.025)	0.7828 (0.092)	0.7822 (0.092)	0.910 (0.051)	0.9046 (0.056)
